# 

**Published:** 1852-10-01

**Authors:** 


### WORKS RECEIVED.

A Commentary of Medical and Moral Life; or, Mind and the Emotions considered, in Relation to Health, Disease, and Religion. By William Cook, M.D., M.R.C.S.L., &c. 1 vol. 8vo. Longman & Co. (In our next.)

On the Diseases of the Bladder. By W. Coulson, Esq., Surgeon to St. Mary's Hospital. 4th edition. Churchill. 1852.

Medical Circular. Nos. 1 to 19.

Iconography; a Tract for the Times. By Vigil.

A Manual of Artistic Anatomy. 1 vol. 8vo. By R. Knox, M.D., F.R.S.E. London: H Renshaw. 1852. (In our next.)

Dietetics of the Soul. (Translated from the German.) By Ernst Von Feuchtersleben, M.D. 1 vol. 12mo. Churchill. 1852.

The Stomach and its Difficulties. By Sir Jas. Eyre, M.D. 1 vol. Churchill. 1852.

On the Anatomy of the Male Urethra, and on the Pathology of Stricture. By H. Hancock, Esq, F.R.C.S., Senior Surgeon to Charing-cross Hospital, Lettsomian Professor of Surgery to the Medical Society of London. London. 1852.—An able, original, and valuable work.

Poems, Essays, and Opinions. By Alfred Bates Richards, Barrister-at-Law, Author of "Crœsus, King of Lydia," a Tragedy, &c. 4 vols. 12mo. 1852.

Rheumatism, Gout, and Neuralgia, as affecting the Head and Ear. By W. Harvey, Esq., Surgeon to the Royal Dispensary for Diseases of the Ear. 1 vol. 8vo. Churchill. 1852.

L'Air Libre et la Vie de Famille dans la Commune, de Gheel. Par le Dr. J. Parigot. Bruxelles. 1852. (In our next.)

The National Temperance Chronicle, for April.

Medical Jurisprudence. By T. R. Beck, M.D., LL.D., and J. B. Beck, M.D. 10th edition. 2 vols. Albany, U.S.A. 1850.

Plea of Insanity. By W. Wood, M.D. 2nd edition. London. 1852.

A Letter to Dr. Lyon Playfair, B.F.R.S., On the Buxton Waters. By W. H. Robertson, M.D., Surgeon and Physician to the Buxton Bath Charity. Pamphlet. A sensible Essay.

A Practical Treatise on Diseases of the Skin. By G. F. Moore Neligan, M.D., M.R.I.A., &c. 1 vol. 8vo. Dublin: Fannin and Co. London: Longman. 1852.

Principles of Human Physiology. By A. B. Carpenter, M.D. 4th edition.

London Labour and London Poor. 1 vol. By H. Mayhew, Esq.

A History of Magic, Witchcraft, and Animal Magnetism. By C. Colquhoun, Esq., author of Isis Revelata. 2 vols. 8vo. London. 1851. (Reviewed in our last No.)

Des Hallucinations, ou Histoire Raisonnée des apparitions, des visions, des songes, de l'extase, du magnétisme et du somnambulisme. Par A. Brierre de Boismont, M.D., &c. Paris. 1852.

Des Principes a suivre dans la Fondation et la Construction des asiles d'aliénés par Max, Parchappe, Inspecteur Général du service des aliénés. Paris: Victor Masson. 1850. In parts. (In our next.)

Etudes Cliniques sur les Maladies Mortales, &c. Par M. Morel, M.D. Paris. 1852. (5 parts received.)

Annals of Pharmacy, and Practical Chemistry. Edited by W. Basticle and W. Dickinson, Members of the Pharmaceutical Society. (1 No. received.)

A Letter to the Rt. Hon. Sir Geo. Grey, Bart., M.P., on Medical Registration, &c. By Emeritus. London. 1852.

On the Fallacies of Homœopathy, &c. By C. H. F. Routh, M.D., &c. London. 1852. (An admirable work, written by a Physician of great promise. We hope to see his great powers of research occupied upon other subjects.)

Bagnes, Prisons, et Criminels. B. Appert. Paris.

The Transactions of the American Medical Association. We have received the 3 vols. of this valuable work. Philadelphia. U.S.A.

Danktoonen Van Meer-en-Berg aan Mr. M. C. Van Hall, voor ziju Keung gedicht Meer-en-Berg, &c. &c. Amsterdam. 1851.

The Transactions of the Association of Medical Officers of Hospitals for the Insane. England.

The Transactions of the Association of Medical Officers of Hospitals for the Insane. America.

Glimpses of an Improved Medical Philosophy. By Ed. King, Esq. Surgeon. Philadelphia.

Psychological Speculations. Essay 1, The Theological Department of Psychology. By the Spirit of the Blue Mountains. London.

Report of the alleged Lunatic Friend Society.

A Few Remarks on the Norwich Consultation. By W. Cooper. (For private circulation.)

The Medical Examiner, and Record of Medical Science. Edited by Francis G. Smith, M.D., and J. D. Biddle, M.D., Philadelphia. (Regularly.)

Allgemeine Zuschrif für Psychiatrie und Psychisch-gerichtliche Medicin, &c. &c. unter der Redaction Von Dameron, Flemming und Roller. Berlin (Regularly.)

The Trial of W. Freeman, for the Murder of John G. Van Nest. Reported by B. F. Hall, Counsellor-at-Law. Auburn.

### JOURNALS IN EXCHANGE.

The Medical Times and Gazette (regularly).

The British and Foreign Medico-Chirurgical Review (regularly).

The Ecclesiologist (regularly).

Newton's London Journal and Repertory of Arts, Science, and Manufactures (regularly).

The Law Magazine, or Quarterly Review of Jurisprudence (regularly).

The Dublin Quarterly Journal of Medical Science.

The London Journal of Medicine (regularly).

The Provincial, Medical, and Surgical Journal (regularly).

Dublin Medical Press (regularly).

The American Journal of Medical Science. Edited by Dr. Hays, Philadelphia (regularly).

The American Journal of Insanity (occasionally). We are often obliged to refuse this journal, in consequence of the heavy postage.

Dr. Ranking's Half-yearly Abstract of the Medical Sciences (regularly).

The Theological Critic. Edited by the Rev. T. K. Arnold, M.A. (regularly).

The Monthly Journal of Medical Science, Edinburgh (regularly).

The Morning Side Mirror. Printed at the Asylum Press, Morning Side (regularly).

Bericht über die Leistungen in der Psychiatrick. Von Dr. H. Lachn, Zweitem Arzte der prov.

Tuenanstalt Cei Halle.

Annales Medico-Psychologiques. Par MM. Les Docteurs Baillanger, B. de Boismont, et Cerise. Paris (regularly).

The Charleston Medical Journal and Review (irregularly, in consequence of the heavy postage occasionally charged).

---

### PSYCHOLOGICAL APPOINTMENTS.

Dr. Hood has been elected physician to Bethlem Hospital, and Mr. Helps has been appointed apothecary to the same institution.

D. F. Tyerman, Esq., Medical Superintendent of the Cornwall County Lunatic Asylum, has been elected resident medical officer of the Male department of Colney Hatch, vice Dr. Hood. Salary, 200*l.* per annum, with a furnished residence, and 100*l.* in lieu of board, with coals, candles, milk, and vegetables.

J. Millar, Esq., assistant-surgeon of Bethnal Green Asylum, has been appointed resident medical superintendent of the Bucks County Asylum.

---

### BRITISH LUNATIC ASYLUMS.

An elaborate article, based upon the official annual reports of the British County Lunatic Asylums, will appear in the January number.

### INDEX TO VOL. V.

ALDERSON, Baron, his "charge" against private lunatic asylums, 399.
America, lunatic asylums in, 481.
Animal magnetism, 292.
——— irritability of professors of, 320.
——— letters on, by Dr. Williams Gregory, 319.
Asylums, Esquirol's opinion of, 289.
——— for the insane, in the organization of, 519.
——— for lunatics at Erlangen, an account of the, 60.
——— in France, 124, 229, 353.
——— new, in Lancashire, 337.
——— private character of, 159.
——— public and private, 343.
Asylum, treatment in, compared with home, 524.
Bacon, Lord, his metaphysical studies, 90.
Beggs, Thomas, on juvenile depravity, 161.
Bergmann, Dr. G. H., on different organs of the brain and their functions, 56.
Bethlem Hospital, alteration of the medical organization of, 407.
——— commission of inquiry into the management of, 407.
——— duties and position of medical superintendent of, 407.
——— election of medical superintendent of, 413.
——— exempt from Lord Shaftesbury's Act, 407.
Biography of men of genius psychologically considered, 3.
Blakey, Robert, on the history of the philosophy of the mind, 82.
Blanchard, J. Laman, character of his illness, 268.
Blood, influence of the state of the, on sleep, 75.
Body, connexion of the, with the mental functions, 224.
Body, the, how affected by insanity, 173.
Bone, S. Vallis, Esq., Cases in Lunacy reprinted by, 531.
Braid, James, his observations on the history of magic, witchcraft, and animal magnetism, 292, 316.
Brain, organs of the, and their functions, by Dr. G. H. Bergmann, 56.
——— physiology and pathology of the, by Joseph Lalor, M.D., 139.
——— the relation of the faculty of speech to the interior lobes of the, by Dr. W. Nasse, 56.
Brierre de Boismont, M., his writings on psychology, 461.
Bucknill, John Charles, M.B., on the classification and management of criminal lunatics, 103.
Burton on melancholy, 260.
Butler's, the, insanity, 282.
Carleton, Mrs., inquiry into the nature and effects of the nervous influence, by, 221.
Carpenter, W. B., on principles of physiology, 41.
Coachman's, the, insanity, 282.
Chloroform and ether, use of, in cases of mania, 63.
Christianity, the influence of its establishment in philosophical inquiries, 87.
Cleanliness, absence of, in some patients, 395.
Colney-Hatch Asylum, cost of construction of, 412.
——— first medical report of, 410.
Colquhoun, J. C., on magic and animal magnetism, 292.
Commissioners in lunacy, sixth annual report of, 336.
Commissions in lunacy, difficulty in superseding them in England, 470.
"Crib bedstead," description of, 398.
Court fools, the history of, by H. F. Flögel, 64.
Crime and insanity, similarity between, 181.
Crime and its punishment in the United States, 527.
Crime, induced by the over-indulgence of the animal passions, 201.
——— statistics of, 445.
——— prevention of, by M. D. Hill, Q.C., Recorder of Birmingham, 153.
Criminal cases, plea of insanity in, 103.
Criminal children, the education of, by the late Joseph Fletcher, Esq., 528.

Criminal lunatics, custody of, 341.
———— on the classification and management of, by J. C. Bucknill, M.B., 103.
———— suggested treatment of, 114.
———— on the management of, by William Wood, M.D., 103.
Criminal mind, analysis and motive and effect, 417.
Criminals, uniform type of, 417.
Cummings, Mrs. Catherine, commission of lunacy on, 256.
Dickson, T., on public asylums for the insane of the middle and higher classes, 343.
Drunkenness, effect of, upon crime, 195.
Education, its effect upon crime, 201.
"Enclosed bed," description of, 397.
England and America, mortality and insanity in separate plan prisons in, 613.
Esquirol, his opinion of asylums, 289.
Ether and chloroform, use of, in cases of mania, 63.
Evidence, medical, in support of plea of insanity, 109.
Ecstatic mania, instances of, 317.
Farm workhouses, 529.
Fibrils, disposition of the ultimate, considered, 47.
Fibrous element of nervous tissue described, 42.
Fletcher, Joseph, Esq., on the education of criminal children, 528.
———— on moral and educational statistics, 161.
Flint, Mr., on crime, 161.
France, asylums in, 124, 229, 353.
———— Code of interdiction in, 466.
Friends, the Society of, insanity among, 183, 186.
Galt, John M., M.D., on the organization of asylums for the insane, 519.
Gelatinous fibres described, 43.
Genius, men of, liable to apoplexy and palsy, 262.
———— eccentricities of, 259.
German psychological literature, 56, 437.
Gregory, Dr. Williams, lectures on animal magnetism by, 319.
Halle, statistics of the asylum at, 444.
Health and disease, effects of, in the magnetic atmosphere in which we live, 223.
Hemispherical ganglion, its effect on generation and transmission of psychal phænomena, 334.
Hereditary taint, reflections on, 434.
Hill, M.D., Q.C., on the prevention of crime, 153.
Holland, Henry, M.D., on mental physiology, 322.
Homicidal monomania, 415, 437.
Hypochondriasis, symptoms of, 269.
Ideé fixe, 401.
Insane, American institutions for the, 481.
———— effects of associating with the, 286.
———— responsibility of the, 119, 123, 174.
———— the medico-legal question of the confinement of the, 523.
Insanity, caused by the over-indulgence of the animal passions, 187.
———— definitions of, by different judges, 103.
———— early symptoms of, 10.
———— incipient, 261.
———— in Millbank prison, Dr. Baly's statistics of, 452.
———— its causes, prevention, and cure, by Joseph Williams, M.D., 276.
———— statistics of, in America, 482.
———— the plea of, in criminal cases, 103.
———— the supposed increase of, by Edward Jarvis, M.D., 257.
———— what evidence will support a plea of, 107.
———— per centage of, in the different prisons of England and America, 515.
Insenescence of the intellectual principle, considered with regard to medical jurisprudence, 390.
Insomnium, borne by some minds with comparative impunity, 70.
———— effects of, 69.
———— often found in nervous childbed women, 72.
Inspectors of prisons of Great Britain, seventh report of the, 457.
Interdiction, code of, in France, 460.
Intellectual life, considered in its different stages, 480.
Intellectual faculties, premature development of, 6.
Intellectual principle, its growth not decline, but onward progress, 387.
Intemperance a predisposing cause of insanity, 186.
Jobard, the homicide, case of, 423.
Johnson's, Dr., opinion of the "metaphysical poets," 15.
Judges, opinions of the, with reference to medical witnesses, 110.
Justice, administration of, in criminal cases, 121.
Juvenile depravity, an inquiry into the extent and causes of, by Thos. Beggs, 161.
Kant, on metaphysics, 98.
Kirkes', W. S., hand-book of physiology, 41.
Lalor, Joseph, M.D., on the physiology and pathology of the brain, 139.
Law, defective state of the, as to the property of lunatics, 341.

Life, zoonomic and intellectual, compared, 480.
Literary life, the wear and tear of, 1, 27.
Lordat, M., his definition of life, 471.
——— his lectures on mental dynamics, 384, 471.
Lucid intervals, deceptive, 34.
Lunacy, answer of lunatic's committee—evidence of lunatic's administratrix, 538.
Lunacy, commission of, on Mrs. Catharine Cumming, 256.
——— costs of an inquisition in, when it is superseded, 534.
——— in England, state of, 336.
——— jurisdiction of the lords justices, 542.
——— legal cases in, 531.
——— the law of, 460.
Lunatic, admissibility of evidence of a, 340.
——— arrangements for the funeral of a deceased, 543.
——— conveyance by a, supported, notwithstanding the lunacy, 531.
——— defendant, answer and defence of a, 541.
——— irresponsibility of, 418.
——— patients, aggregate number of, in 1851, 337.
Lunatics, criminal, suggested treatment of, 114.
Lunatic's estate, allowance made out of, to members of the family, 544.
Madness, imaginative brains liable to, 72.
——— incipient, peculiarities of, 73.
Magic, witchcraft, and animal magnetism, observations on the history of, by James Braid, 292, 316.
Magicians and priests, powers of the ancient, 309.
Man, comparison of infancy of, with that of animals, 476.
Manchester, royal lunatic asylum at Cheadle, 338.
Materialists, objections to the views of, 475.
Medical ethics, 390.
——— as affected by comparative anatomy, 391.
——— compared with rational philosophy, 394.
Medical evidence in criminal cases, 107.
Medical nomenclature, discrepancy of, 323.
Medical officer of a lunatic asylum, position of, 486.
——— selection of, 521.
Medical superintendent of Bethlem Hospital, qualifications for, 413.
Medical witness, duty of, 113.
Medicine, the science of, mental dynamics in relation to, 384, 471.
Melancholia and suicidal monomania, 267.
Melancholy, anatomy of, by Burton, 260.
Memory, ill effects of over-exertion of the, 330.
——— the pathology of, 328.
Men of genius, their studious habits, 9.
Men, natural passions of, 161.
Mental dynamics in relation to the science of medicine, 384, 471.
Mental diseases not to be compared with bodily diseases, 33.
Mental philosophy in Scotland, 83.
——— in the 15th century, 86.
——— little studied in this country, 82.
Mental physiology, by Henry Holland, M.D., 322.
Mental progression, evidence of, 3.
Mesmerism, essence of, in the patient, not in the operator, 325.
"Metaphysical Poets," Dr. Johnson's opinion of the, 15.
Metaphysicians, the Scottish school of, opposed by Lady M. Shepherd, 100.
Millbank Prison, report of, 451.
Mind, history of the philosophy of the, by Robert Blakey, Esq., 82.
——— the comprehensiveness of the, 2.
——— the overworked, 257.
Monomania, a curious case of, described, 401.
——— analysis of, 419.
Monro, H., M.B., on the reform in private asylums, 343.
Moral and educational statistics, by J. Fletcher, Esq., 161.
Mortality and insanity in separate plan prisons in England and America, 483.
Muscular action, effects of attention on, 325.
Nasse, Dr. W., on the relation of the faculty of speech to the interior lobes of the brain, 56.
Nerve fibres, central and peripheral terminations of, considered, 46.
Nerve fibrils, cerebro-spinal, appearance of the, 43.
Nerves of sensation, how stimulated, 51.
——— laws peculiar to, 52.
Nervous action, different temperaments of, 225.
Nervous influence, effect of, on the moral and intellectual character, 225.
——— inquiry into the nature and effects of the, by Mr. Carleton, 221.
Nervousness, produced by irregular living, 273.
Nervous tissue, fibrous elements of, described, 42.

Nervous tissue, the structure and functions of, 41.
———— the vesicular or corpuscular structure of, described, 44.
Non-restraint system in Germany; 443.
" Odyle force," description of, 312.
Opium, use of, in different forms of insanity, 439.
Paralysis, Dr. Sholz on, 442.
Parkhurst Prison, report of, 448.
Parrish, Dr., remarks by, on insanity in England and America, 403.
Penal code in Pennsylvania, mildness of, 403.
Pennsylvania, description of the separate systems of punishment in, 404.
Philosophy, characteristics of the German school of, 100.
Pyschological literature, German, 56.
———— science, progress of, 302.
Psychology of epochs, the, 208.
Physical discovery, modern, 223.
Physical School of Paris, past and present, reviewed, 384, 386.
Physiology, hand-book of, by W. J. Kirkes, M.D., and J. Paget, 41.
Physiology, principles of, by W. B. Carpenter, M.D., 41.
Pentonville prison, report of, 446.
Prichards, Dr., definition of monomania, 420.
Prisons, moral and mental condition of, 445.
Prisons of America, per-centage of insanity in the different, 116.
——— of England, per-centage of insanity in the different, 515.
——— of Great Britain, seventeenth report of the inspectors of, 457.
——— separate plan, mortality and insanity in, England and America, 513.
Quakers, insanity among, 183, 186.
Reason and instinct, difference between, 190.
Reid, Dr., founder of the Scottish school of psychology, 97.
Saxon metaphysics, 89.
Scenery, influence of, on the poetic character, 18.
Schools of Paris and Montpellier contrasted, 384.
Scott, Sir W., description of his illness, 264.
Scrofula and consumption, produced by solitary confinement, 406.
Second childhood, refuted, 387.
Self-imposed restraint as a measure of precaution, 421.
Self-indulgence beyond necessary limits opposed to reason, 179.
Separate confinement injurious to the coloured race, 406.
Silent system of punishment in Pennsylvania described, 404.
Shepherd, Lady M., her writings against the Scottish school of metaphysicians, 100.
Slaves, infrequency of mental alienation among, 214.
Sleep, affected by the state of the blood, 75.
—— importance of, to insane patients, 395.
—— prevented by anxiety of mind, 70.
—— the only mental prophylaxis of mental decadence, 68.
—— the pathology of, 67.
—— the proximate cause of, 74.
Sleeplessness, remedies for, 78.
———— the cause and effect of insanity, 69.
Sleep and death, analogy between, 68.
Solitary confinement, comparative effect of long and short sentence, 514.
———————— graduated scale of, adapted to the condition of the individual, 518.
———————— scrofula and consumption produced by, 406.
Sonnenstein, report from the public asylum at, 442.
Sorcery in ancient times, 297.
Southey, the last days of, 1.
——— his nervous temperament, 25.
——— his early dread of insanity, 11, 26.
——— not a psychological poet, 17.
Spinoza, his opinions on metaphysical philosophy, 91.
State asylums, 115, 119.
Studious men liable to cerebral diseases, 262.
Thought and imagination, connexion between, 318.
Treatment of the insane, the "cottage system," 288.
United States, ratio of crime and punishment in the, 527.
Vital force, its rise and decline, 387.
Walking exercise recommended to the student, 275.
Williams, Josh., M.D., on insanity, 276.
Winslow, Dr. Forbes, his researches on crime and its punishment, 403.
Wood, William, M.D., remarks on the plea of insanity and on the management of criminal lunatics, 103.
Zoonomic life, considered in its different stages, 477.

LONDON
SAVILL AND EDWARDS, PRINTERS,
CHANDOS STREET.

